# Identification of a *PATL2* missense variant (c.877G>T) disrupting canonical splicing and contributing to female infertility

**DOI:** 10.3389/fgene.2025.1611138

**Published:** 2025-07-09

**Authors:** Hongyan Li, Yue Lin, Weixu Ma, Ting Yu, Lingfeng Dong, Yankun Chen, Shuming Fan, Guoqun Luo, Jingwen Zhang, Ge Song

**Affiliations:** Affiliated Foshan Maternity and Child Healthcare Hospital, Guangdong Medical University, Foshan, Guangdong, China

**Keywords:** infertility, oocyte maturation disorders, *PATL2*, missense variant, aberrant splicing

## Abstract

**Background:**

*PATL2* deficiency is a significant cause of female infertility. Although multiple *PATL2* missense variants have been reported in prior studies, a number of these variants remain classified as variants of uncertain significance (VUS).

**Methods:**

We present a patient of primary infertility characterized by oocyte maturation disorders and fertilization failure. Comprehensive genetic analysis was conducted through whole-exome sequencing (WES) to identify pathogenic variants, followed by Sanger sequencing for familial co-segregation analysis. Reverse transcription (RT-PCR), cDNA sequencing and quantitative RT-PCR were performed to validate the effect of the variant on pre-mRNA splicing.

**Results:**

We identified compound heterozygous variants in the *PATL2* gene by WES: a pathogenic splice-site splicing variant (c.223-14_223-2del) and a missense variant (c.877G>T) initially classified as a VUS. Sanger sequencing confirmed that the proband carried biallelic variants, whereas her sisters with either wild-type genotypes or a single heterozygous variant exhibited normal fertility, supporting the co-segregation of the identified variants. Critically, RNA assays demonstrated that the missense variant c.877G>T disrupts canonical splicing of *PATL2*, resulting in exon 12 skipping.

**Conclusion:**

This study provides the first experimental evidence that a *PATL2* missense variant (c.877G>T) can exert its pathogenicity through aberrant splicing, supporting its pathogenic reclassification and elucidating a genotype-phenotype correlation for *PATL2* missense variants through functional assays.

## 1 Introduction

Infertility is an important issue in human reproductive health. According to a newly published report by WHO, approximately 17.5% of the adult population experiences infertility. With the widespread application of assisted reproductive technology, including *in vitro* fertilization (IVF) and intracytoplasmic sperm injection (ICSI), most infertile couples are able to have their own babies. However, recurrent IVF/ICSI failure still persists in some patients. Such failures can be result from problems related to oocyte production which can be caused by various factors, including defects in gonadal differentiation and ovary development, oocyte maturation and gamete recognition, or early embryonic development (Solovova and Chernykh, 2022). Oocyte maturation involves the stages from germinal vesicle (GV) to metaphase I (MI) and ultimately to metaphase II (MII) oocytes, is a fundamental prerequisite for reproduction (Pei et al., 2023). Oocyte maturation disorder is a rare syndrome characterized by the repeated production of a majority of immature oocytes, causing primary infertility, repetitive production of immature oocytes, inability of *in vitro* maturation (IVM) to stimulate maturation, and recurrent fertilization failure after IVF/ICSI (Beall et al., 2010). Oocyte maturation is a well-organized complex process. In recent years, emerging evidence highlights the pivotal role of genetic factors in oocyte maturation disorder, fertilization failure, early embryonic development arrest, revealing multiple disease-causing genes (Fei and Zhou, 2022).

The *PATL2* gene encodes Protein PAT1 homolog 2, an RNA-binding protein, is more highly and specifically expressed in human germinal vesicle, metaphase I, and polar body I oocytes than in various somatic tissues. PATL2 is highly expressed before germinal vesicle breakdown (GVBD). As the oocyte matures, PATL2 is gradually degraded in the cytoplasm of oocytes (Chen et al., 2017). PATL2 acts as a translational repressor during oocyte maturation (Nakamura et al., 2010; Christou-Kent et al., 2018), is regulated maternal mRNA expression in immature oocytes. Variants in human *PATL2* gene lead to oocyte/zygote/embryo maturation arrest-4 (OZEMA4, OMIM: 617743). These findings indicate that temporal control of PATL2 expression levels is critical for the normal maturation of oocytes.

In the present study, we identified compound heterozygous variants of *PATL*2 in a patient with primary infertility: a pathogenic splice-site variant (c.223-14_223-2del; p.Arg75Valfs*21) and a missense variant (c.877G>T; p.D293Y) initially classified as a VUS. We first demonstrated that the missense variant c.877G>T disrupted canonical splicing, inducing exon 12 skipping in the mRNA transcript and producing an in-frame 18-amino acid deletion (p.293_310del) in the N-terminal PAT1 domain. Given the critical role of the PAT1 domain in RNA binding (Nakamura et al., 2010), this deletion might potentially impair the protein’s function. Our findings offered evidence for re-evaluating the pathogenicity of the c.877G>T variant, and provided new insight into the pathogenic mechanism of missense variants in the *PATL2* gene.

## 2 Methods

### 2.1 Evaluation of oocyte and embryo phenotypes

Oocytes were obtained from the proband and a normal individual undergoing *in vitro* fertilization (IVF) or intracytoplasmic sperm injection (ICSI). Morphological assessments of oocyte maturation, fertilization, and embryonic development were performed using time lapse imaging. The study was approved by the Medical Ethics Committee of Foshan Maternity and Child Healthcare Hospital, Guangdong Medical University (No. FSFY-MEC-2025-028). The participants provided their written informed consent to participate in this study.

### 2.2 Whole-exome sequencing and pathogenicity analysis

Genomic DNA (gDNA) was extracted with QIAamp DNA Blood Mini Kit (QIAGEN, Hilden, Germany) from peripheral blood of proband and other family members using standard methods. Candidate variants were identified by whole-exome sequencing (WES). Briefly, DNA was fragmented and processed using the NadPrep DNA Universal Library Preparation Kit (Nanodigmbio, Nanjing, China) for whole-genome and exome library construction. Whole-exome enrichment was performed with the Exome Plus Panel v2.0 (Nanodigmbio, Nanjing, China) targeting exon and known pathogenic regions. Libraries were sequenced on the DNBSEQ-T7 platform (MGI Tech, Shenzhen, China). The sequencing data were converted to FASTQ format. Clean reads were aligned to the UCSC hg19 reference genome using BWA software, with duplicate reads removed via Picard. Variants, including SNVs and InDels, were identified using GATK HaplotypeCaller. Variants were annotated with ANNOVAR. The online software SpliceAI, dbscSNV, MaxEntScan, SPiCE, CADD splice were used to predict the influence on the splicing. Pathogenicity analysis of variants was performed according to the ACMG/AMP practice guidelines.

### 2.3 Variant validation and co-segregation analysis

Sanger sequencing was used to verify the candidate variants in the proband and perform segregation analysis in the other family members. The following Sanger sequencing primer pairs were used: PATL2-c223-F/R and PATL2-c877-F/R.

### 2.4 RT-PCR, cDNA sequencing and quantitative RT-PCR

Total RNA was isolated from the peripheral blood leukocytes of the proband and control individuals using TRIzol reagent (Invitrogen, MA, United States). RNA was reverse-transcribed using a TaKaRa PrimeScript reagent kit (TaKaRa, Dalian, China) according to the manufacturer’s protocol. To investigate the aberrant splicing of the variant of *PATL2*, we amplified *PATL2* cDNA spanning exons 10 to 14 in the proband and a normal control individual (NC) using primers PATL2-E10-F and PATL2-E14-R. Then, the obtained PCR products were analyzed by gel electrophoresis on a 1% agarose gel. Individual bands were excised from the gel, eluted using the Gel Extraction Purification Kit (QIAGEN, Hilden, Germany) and the further Sanger sequencing was performed on the cDNA purified product. Quantitative RT-PCR (qPCR) was conducted using SYBR Premix Ex Taq (Takara, Dalian, China) according to the manufacturer’s protocol. GAPDH served as an endogenous control. The 2^−ΔΔCt^ method was used to analyze the relative gene expression. All sequencing and qPCR primers used in this study are listed in Supplementary Table S1.

## 3 Clinical description and results

### 3.1 Clinical characterization

**FIGURE 1:**
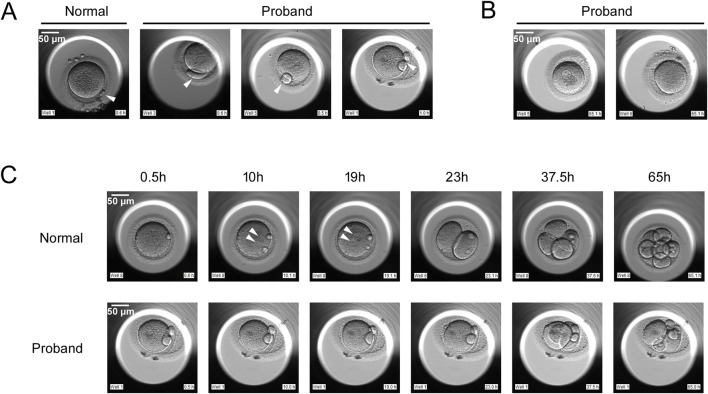
Phenotypes of oocytes from normal individual and the proband with *PATL2* variants. **(A)** The morphology of metaphase II (MII) oocytes from a normal individual and the proband. The proband had MII oocytes with abnormal polar body, as indicated by the arrows. **(B)** Oocytes arrested at metaphase I (MI) stage and germinal vesicle (GV) stage after 65 h *in vitro*. **(C)** Development of fertilized eggs from a normal individual and the proband. In the proband’s MII oocytes subjected to ICSI, no pronucleus formation was observed, and abnormal cleavage was present. The arrows indicate the pronucleus. Scale bar, 50 μm.

The proband, a 30-year-old female, and her 28-year-old husband had been diagnosed with primary infertility for 1 year. Both had normal karyotypes. Following two unsuccessful intrauterine insemination (IUI) cycles, the couple underwent two consecutive IVF/ICSI cycles, neither of which yielded viable embryos. In these two cycles, we used a time-lapse embryo incubator for cultivation and imaging, with images captured every 15 min for 7 consecutive days of continuous observation. In the first cycle, 16 oocytes were retrieved. Then IVF fertilization was conducted, but total fertilization failure occurred. After 16-18 h *in vitro*, 1 germinal vesicle (GV) stage oocyte, 2 metaphase I (MI) stage oocytes, and 13 metaphase II (MII) oocytes were observed. Strikingly, all MII oocytes displayed morphological abnormalities, notably enlarged or double polar bodies (Figure 1A). Subsequently, Late rescue ICSI was conducted on 8 MII oocytes, but no pronuclei formation was observed, 1 oocyte showed abnormal cleavage. Eventually, no embryo was obtained, and the cycle was canceled. In the second cycle, 18 oocytes were retrieved, 8 at the GV stage, 4 at the MI stage, and 6 at the MII stage. Similar to the first cycle, all MII oocytes again exhibited polar body defects (Figure 1A). ICSI was performed on 6 MII oocytes, but no pronuclei formation was observed on D1. 4 oocytes showed abnormal cleavage on D2 and D3 (Figure 1C). Eventually, no embryos were obtained, and the cycle was canceled. Notably, 6 oocytes were arrested at the GV stage, 4 oocytes were arrested at the MI stage after 65 h *in vitro* (Figure 1B). The proband has five sisters and two brothers range from 20 to 40 years old, all of whom exhibit normal fertility without reproductive complications.

### 3.2 Identification of variants in *PATL2* gene

WES was performed to elucidate the genetic basis of infertility in the proband (individual II-4). Bioinformatic analysis identified two heterozygous variants in the *PATL2* gene (NM_001387263.1): a splicing variant c.223-14_223-2del (p. R75Vfs*21) and a missense variant c.877G>T (p.D293Y). These variants may be responsible for the phenotypes of the proband. No pathogenic variants were detected in other known female infertility or oocyte development-associated genes. The c.223-14_223-2del variant was classified as pathogenic based on prior literature and database annotations (Chen et al., 2017; Huang et al., 2018). While the c.877G>T variant was initially classified as a VUS according to ACMG/AMP guidelines (PM2_Supporting, PM3_Supporting, PP3), the supporting evidence is as follows: 1. The c.877G>T variant has an allele frequency of 0% in population databases and has been undetected in all major control cohorts (e.g., gnomAD, ExAC, 1,000 Genomes, NHLBI ESP) (PM2_Supporting); 2. In a female infertility patient, it cannot be determined whether this variant and another variant c.223-14_223-2del (a known pathogenic variant) are in cis or trans configuration (PM3_Supporting) (Wu et al., 2019); 3. Multiple *in silico* prediction tools (e.g., SpliceAI, dbscSNV, MaxEntScan, SPiCE, CADD splice) suggest that this variant may disrupt mRNA splicing (PP3).

**FIGURE 2:**
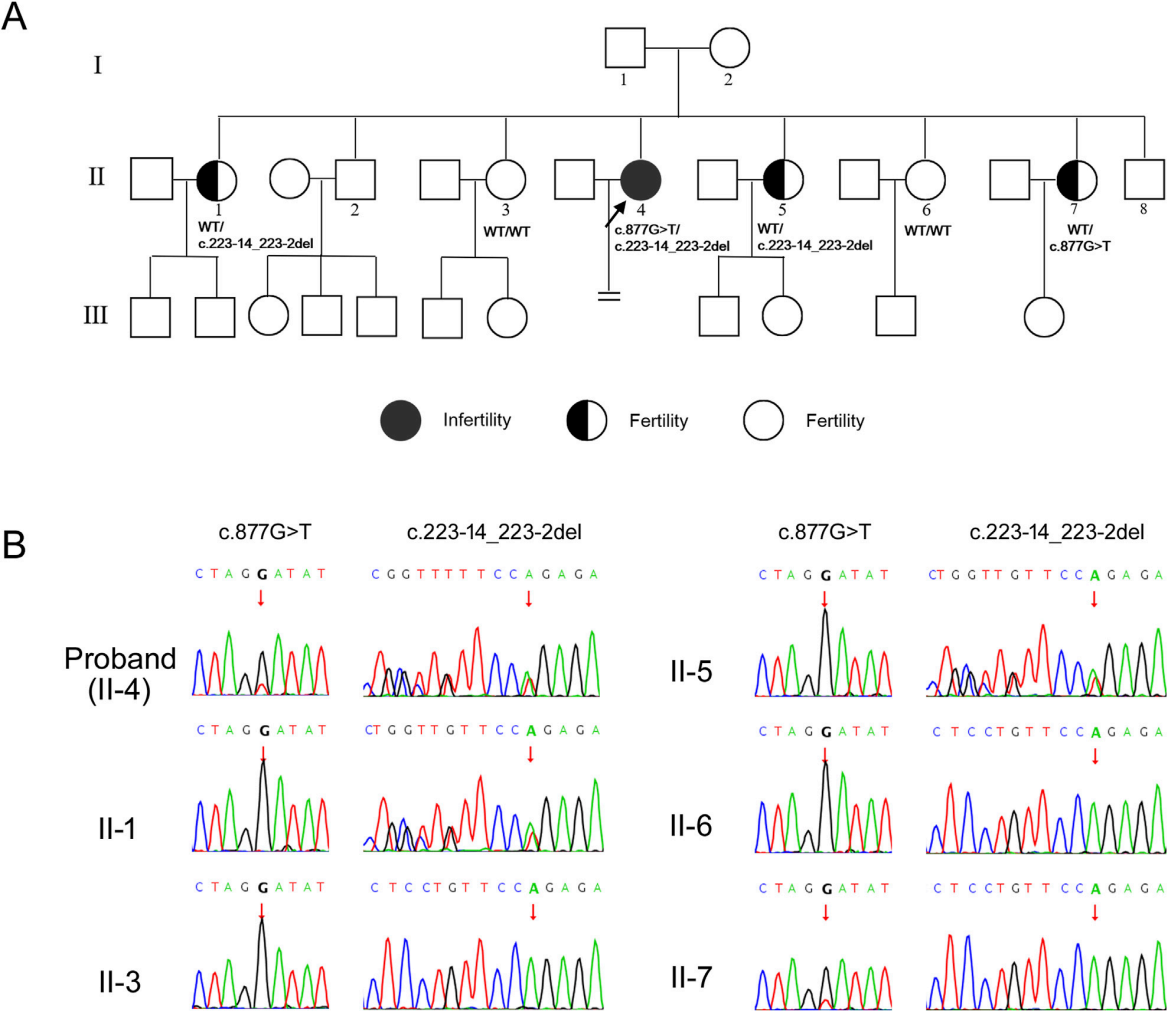
Identification of variants in *PATL2* gene. **(A)** Pedigrees of the affected family. Black circle represents affected individuals. Half black circles represent carriers. Clear circles represent unaffected individuals. The black arrow indicates the proband. The “ = ” sign indicates infertility. Corresponding Sanger sequencing results are presented below the family members. **(B)** Sequencing chromatograms of family members. The figure illustrates carrying status of the variant c.877G>T and c.223-14_223-2del among the family members. The arrows in the chromatograms indicate the positions of the variants.

Then, Sanger sequencing was used to verify the candidate variants in the proband and to perform segregation analysis among other family members (Figure 2A). The Sanger sequencing results indicated that the proband’s two sisters (II-3, II-6) exhibited wild-type genotypes (WT/WT) at both loci, two other sisters (II-1, II-5) were heterozygous carriers of the c.223-14_223-2del variant, the youngest sister (II-7) was heterozygous carriers of the c.877G>T variant (Figure 2B). All the sisters of proband retained fertility, consistent with an autosomal recessive inheritance pattern and supporting co-segregation of the compound heterozygous variants with the infertility phenotype. Following validation, the variant c.877G>T lies in trans to the pathogenic variant c.223-14_223-2del, meeting PM3 criteria for pathogenic evidence. Combining segregation data from affected and unaffected individuals yielded a LOD score of 0.62, meeting PP1 criteria for pathogenic evidence (Oza et al., 2018).

### 3.3 Confirmation of the aberrant splicing

**FIGURE 3:**
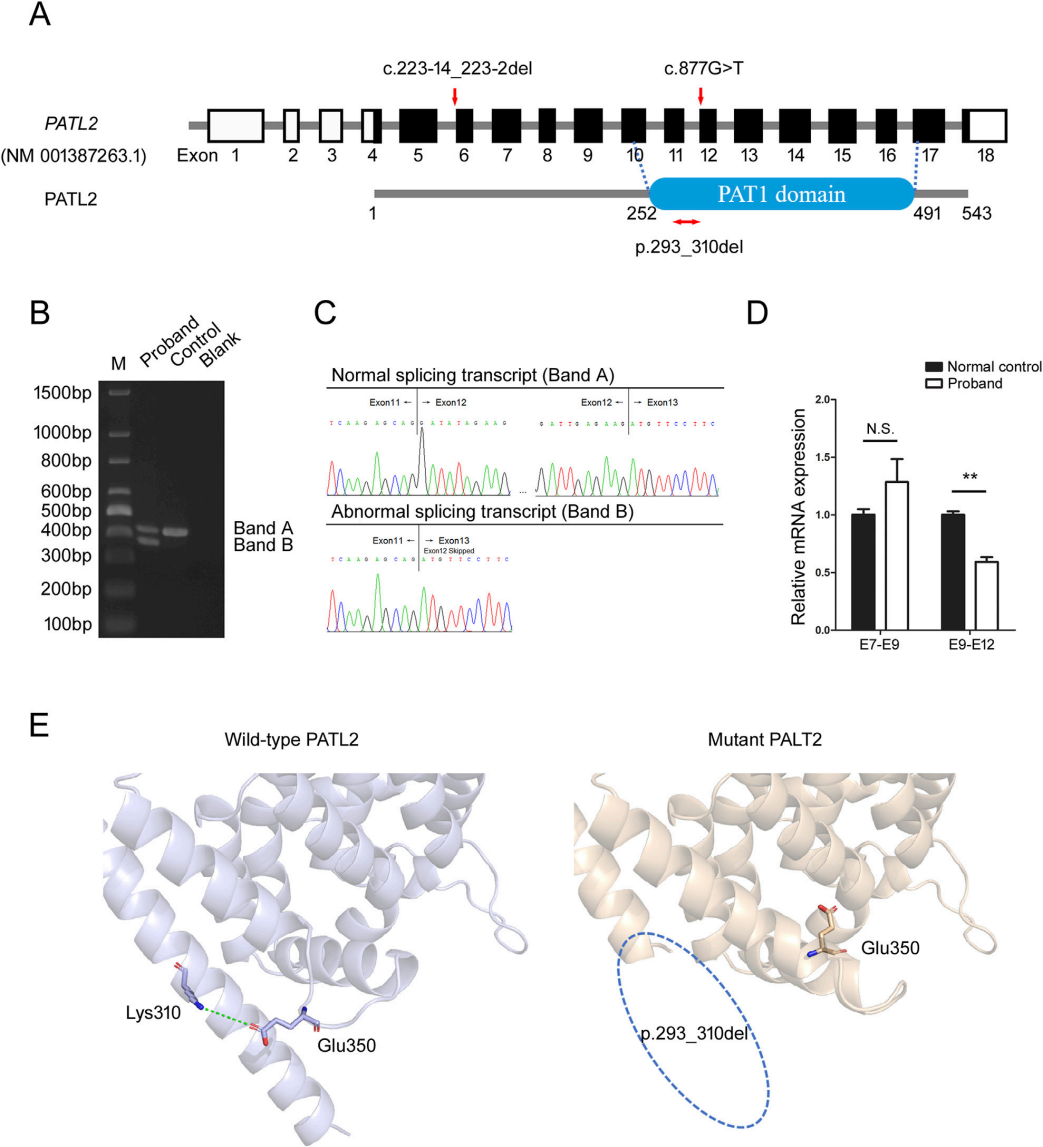
Identification of aberrant splicing in *PATL2*. **(A)** The positions of variants and functional domains are indicated in the gene structures. The missense variant c.877G>T leads to in-frame deletion (p.293_310del) in *PATL2* is marked in red. **(B)** Agarose gel electrophoresis of RT-PCR products reveals two distinct amplification products: Band a corresponds to the wild-type transcript (normal splicing), while Band b represents the aberrant splicing isoform. M means the DNA marker. **(C)** Chromatograms demonstrate the precise splicing patterns corresponding to the RT-PCR products shown in **(B)**, confirming the existence of both normal and aberrant transcripts. **(D)** Quantitative RT-PCR analysis of relative *PATL2* mRNA expression levels in the proband and normal female controls (NC). E7-E9 represents amplification of the exon7-exon9 region; E9-E12 represents amplification of the exon9-exon12 region. N.S.: Not Significant (P > 0.05), **P < 0.01 (t-test), Error bars represent mean ± SEM (n = 3). **(E)** Structural modeling of wild-type and mutant PATL2 proteins using AlphaFold3. The wild-type PATL2 is presented in gray, while the mutant PALT2 is presented in wheat color. Blue dotted circle: a segment of the α-helix within the PAT1 domain of the mutant PALT2 is absent.

The missense variant c.877G>T was located on the first base of exon 12 in the *PATL2* gene (NM_001387263.1), adjacent to the splicing acceptor site of intron 11-a region critical for splicing fidelity (Figure 3A). Multiple *in silico* prediction tools (e.g., SpliceAI, dbscSNV, MaxEntScan, SPiCE, CADD splice) suggest that this variant may disrupt mRNA splicing, SpliceAI suggested the score of acceptor loss was 0.43 (threshold ≥ 0.2), indicated that the variant (c.877G>T) was likely to change the acceptor spot for mRNA splicing. To assess the impact of the variant on *PATL2* transcript processing, total RNA was isolated from the proband and normal control individual. The RT-PCR amplification of *PATL2* transcripts spanning exons 10-14 revealed two distinct bands in the lane of proband (bands A and B), contrasting with a single product (band A) observed in the lane of control sample (Figure 3B). Sanger sequencing of gel-purified products demonstrated that band A corresponded to the canonical splicing pattern (exon11-exon12-exon13), but band B was an aberrant splicing band which skipped complete exon12 (exon11-exon13) (Figure 3C). Exon12 contain 54 base pair. So, this splicing defect led to 18-amino acid in-frame deletion (p.293_310del) in N-terminus of PAT1 domain of PATL2 (Figure 3A). We subsequently used qPCR to investigate the abundance of aberrant splicing variants at the mRNA level. Primers targeting exon7-exon9 (shared by both alleles) were used to evaluate the total expression of *PATL2*, while mutant-specific primers (forward: upstream of the deletion region [exon 9]; reverse: within the deletion region [exon12]) specifically amplified the other allele. The results showed that the total *PATL2* mRNA level in the proband was slightly increased compared with normal controls, but the difference was not statistically significant. The mRNA level of the exon 9-exon12 region in the proband was approximately half that of normal control (Figure 3D).These findings indicated that aberrant splicing may not result in degradation of *PATL2* mRNA. Collectively, RNA assays demonstrated that the c.877G>T variant results in truncation of <10% of the full-length PATL2 protein, meeting the criteria for PVS1_Moderate according to the ACMG/AMP guidelines.

To illustrate the structural location of the deleted amino acids within the protein, we employed AlphaFold3 (Abramson et al., 2024) for structural modeling of wild-type and mutant PATL2 proteins, which revealed that this variant caused a deletion of an alpha-helix in the PAT1 domain. Further analysis demonstrated that a critical salt bridge interaction between Lys310 and Glu350 in the wild-type protein was disrupted by the Asp293-Lys310 deletion (Figure 3E). While these protein structure predictions have not been experimentally validated and serve only as supporting evidence, they can provide valuable insights for subsequent research.

## 4 Discussion

The *PATL2* gene encodes an RNA-binding protein known as Protein PAT1 homolog 2 that regulates maternal mRNA homeostasis. P100 in *Xenopus* oocytes is the orthologue of human PATL2, which was regarded as a mRNA-binding protein (mRNP) associated with other mRNPs, such as Xp54, xRAP55, and CPEB. plays a role in regulating the translation of specific maternal mRNAs required for the progression of *Xenopus* oocyte maturation (Nakamura et al., 2010). Consistent with this role, Patl2 knockout mice exhibit oocyte and zygote morphological and developmental defects. Alongside significant downregulation of gene expression associated with oocyte maturation, including CDC25a and SohIh2 in PATL2-absence mouse oocytes (Christou-Kent et al., 2018). In humans, biallelic *PATL2* variants, including homozygous or compound heterozygous variants lead to infertility due to oocyte germinal vesicle (GV) arrest or MI arrest, fertilization failure, and early embryo developmental arrest (Huo et al., 2022; Ye et al., 2024). All of these researches indicated that the *PATL2* play a pivotal role in oocyte maturation.

In this study, we identified compound heterozygous *PATL2* variants in a patient with primary infertility due to oocyte maturation disorders and fertilization failure: a pathogenic splice-site variant (c.223-14_223-2del, p.R75Vfs*21) and a missense variant (c.877G>T, p.D293Y). The latter was only reported in one patient, and the patient carried heterozygous variants c.877G>T and c.223-14_223-2del with an unknown inheritance pattern (Wu et al., 2019). Thus, the pathogenicity of the variant c.877G>T was not well defined. To understand the molecular pathogenesis of the missense variant c.877G>T, we performed RNA assay and confirmed that the c.877G>T variant caused aberrant splicing of the *PATL2* transcript characterized by exon 12 skipping (Δ54 bp), resulting in an in-frame 18-amino acid deletion (p.293_310del) in the N-terminus of PAT1 domain. The PAT1 domain is a critical functional domain responsible for RNA binding. Another variation that is different at the DNA level but leads to the same protein-level deletion has also been reported. The canonical splice-site variant *PATL2*: c.877-1G>A was previously reported as pathogenic in patients with primary infertility due to oocyte maturation arrest (OMA). In two unrelated OMA patients, this variant was experimentally proven to cause exon 12 skipping, resulting in an in-frame 18-amino acid deletion (p.293_310del) within the N-terminal PAT1 domain (Sun et al., 2022; Zhu et al., 2022; Zhou et al., 2024). These results also demonstrate that the p.293_310del impairs the function of PATL2, supporting the pathogenicity of the c.877G>T variant. Several *PATL2* missense variants reported in prior studies were documented to either reduce their expression levels or enhance degradation (Liu et al., 2020; Cao et al., 2021; Yu et al., 2024). In this study, we first verified that the missense variant c.877G>T in *PALT2* disrupts canonical splicing, leading to oocyte maturation disorders and fertilization failure.

Owing to the clinical and genetic variability associated with oocyte maturation disorders, WES is considered the primary genetic detection method. WES enables the efficient identification of genetic variants within genes. However, many variants, especially missense variants detected by WES, remain poorly understood and are often classified as “variants of uncertain significance (VUS)”. The mechanisms by which missense variants lead to abnormal protein functions are diverse, making it challenging to definitively assess their pathogenicity. Therefore, experimental validation of the functional impacts of these variants is essential to clarify their clinical relevance. In this study, we provided additional evidence for pathogenicity assessment of the *PATL2* missense variant c.877G>T through experimental validation and familial co-segregation analysis, supporting our proposal to reclassify this variant as “likely pathogenic” according to the ACMG/AMP guidelines (PM2_Supporting, PM3, PVS1_Moderate (RNA), and PP1). These findings offer more information to support genetic counseling and personalized treatment for the proband. Currently, there is no established therapeutic regimen for this disorder, and oocyte donation may serve as a potential fertility option. Collectively, our results underscore the importance of integrating functional assays with genetic analyses to elucidate the pathogenicity of genetic variants and the mechanisms responsible for clinical phenotypes.

## Data Availability

The original contributions presented in the study are included in the article/Supplementary Material, further inquiries can be directed to the corresponding author.

## References

[B1] Abramson J. Adler J. Dunger J. Evans R. Green T. Pritzel A. (2024). Accurate structure prediction of biomolecular interactions with AlphaFold 3. Nature 630 (8016), 493–500. 10.1038/s41586-024-07487-w 38718835 PMC11168924

[B2] Beall S. Brenner C. Segars J. (2010). Oocyte maturation failure: a syndrome of bad eggs. Fertil. Steril. 94 (7), 2507–2513. 10.1016/j.fertnstert.2010.02.037 20378111 PMC2946974

[B3] Cao Q. Zhao C. Wang C. Cai L. Xia M. Zhang X. (2021). The recurrent mutation in PATL2 inhibits its degradation thus causing female infertility characterized by oocyte maturation defect through regulation of the Mos-MAPK pathway. Front. Cell Dev. Biol. 9, 628649. 10.3389/fcell.2021.628649 33614659 PMC7890943

[B4] Chen B. Zhang Z. Sun X. Kuang Y. Mao X. Wang X. (2017). Biallelic mutations in PATL2 cause female infertility characterized by oocyte maturation arrest. Am. J. Hum. Genet. 101 (4), 609–615. 10.1016/j.ajhg.2017.08.018 28965849 PMC5630194

[B5] Christou-Kent M. Kherraf Z. E. Amiri-Yekta A. Le Blévec E. Karaouzène T. Conne B. (2018). PATL2 is a key actor of oocyte maturation whose invalidation causes infertility in women and mice. EMBO Mol. Med. 10 (5), e8515. 10.15252/emmm.201708515 29661911 PMC5938616

[B6] Fei C. F. Zhou L. Q. (2022). Gene mutations impede oocyte maturation, fertilization, and early embryonic development. Bioessays 44 (10), e2200007. 10.1002/bies.202200007 35900055

[B7] Huang L. Tong X. Wang F. Luo L. Jin R. Fu Y. (2018). Novel mutations in PATL2 cause female infertility with oocyte germinal vesicle arrest. Hum. Reprod. 33 (6), 1183–1190. 10.1093/humrep/dey100 29697801

[B8] Huo M. Zhang Y. Shi S. Shi H. Liu Y. Zhang L. (2022). Gene spectrum and clinical traits of nine patients with oocyte maturation arrest. Front. Genet. 13, 772143. 10.3389/fgene.2022.772143 35140748 PMC8819080

[B9] Liu Z. Zhu L. Wang J. Luo G. Xi Q. Zhou X. (2020). Novel homozygous mutations in PATL2 lead to female infertility with oocyte maturation arrest. J. Assist. Reprod. Genet. 37 (4), 841–847. 10.1007/s10815-020-01698-6 32048119 PMC7183019

[B10] Nakamura Y. Tanaka K. J. Miyauchi M. Huang L. Tsujimoto M. Matsumoto K. (2010). Translational repression by the oocyte-specific protein P100 in xenopus. Dev. Biol. 344 (1), 272–283. 10.1016/j.ydbio.2010.05.006 20471969

[B11] Oza A. M. DiStefano M. T. Hemphill S. E. Cushman B. J. Grant A. R. Siegert R. K. (2018). Expert specification of the ACMG/AMP variant interpretation guidelines for genetic hearing loss. Hum. Mutat. 39 (11), 1593–1613. 10.1002/humu.23630 30311386 PMC6188673

[B12] Pei Z. Deng K. Xu C. Zhang S. (2023). The molecular regulatory mechanisms of meiotic arrest and resumption in oocyte development and maturation. Reprod. Biol. Endocrinol. 21 (1), 90. 10.1186/s12958-023-01143-0 37784186 PMC10544615

[B13] Solovova O. A. Chernykh V. B. (2022). Genetics of oocyte maturation defects and early embryo development arrest. Genes 13 (11), 1920. 10.3390/genes13111920 36360157 PMC9689903

[B14] Sun L. Tong K. Liu W. Tian Y. Yang S. Zhou D. (2022). Identification and characterization of a novel homozygous splice site variant of PATL2 causing female infertility due to oocyte germinal vesicle arrest. Front. Genet. 13, 967288. 10.3389/fgene.2022.967288 36072676 PMC9441802

[B15] Wu L. Chen H. Li D. Song D. Chen B. Yan Z. (2019). Novel mutations in PATL2: expanding the mutational spectrum and corresponding phenotypic variability associated with female infertility. J. Hum. Genet. 64 (5), 379–385. 10.1038/s10038-019-0568-6 30765866

[B16] Ye Z. Li D. Niu X. Yang A. Pan Z. Yu R. (2024). Identification novel mutations and phenotypic spectrum expanding in PATL2 in infertile women with IVF/ICSI failure. J. Assist. Reprod. Genet. 41 (5), 1233–1243. 10.1007/s10815-024-03071-3 38536595 PMC11143103

[B17] Yu A. Huang Z. Shi H. Lin Y. Cai X. Ke Z. (2024). Identification of a novel mutation in PATL2 gene associated with the germinal vesicle arrest of oocytes. Biochem. Biophys. Rep. 40, 101886. 10.1016/j.bbrep.2024.101886 39649799 PMC11625204

[B18] Zhou L. Yang M. Mei M. Mai Z. Li X. Deng K. (2024). Exploring the role of non-canonical splice site variants in aberrant splicing associated with reproductive genetic disorders. Clin. Genet. 106 (6), 750–756. 10.1111/cge.14604 39103988

[B19] Zhu L. Yang Q. Jin H. Zhou J. Wang M. Yang L. (2022). Oocyte phenotype, genetic diagnosis, and clinical outcome in case of patients with oocyte maturation arrest. Front. Endocrinol. (Lausanne) 13, 1016563. 10.3389/fendo.2022.1016563 36440233 PMC9684610

