# Supplementary material for: Identification of a PATL2 missense variant (c.877G>T) disrupting canonical splicing and contributing to female infertility
Source: Front Genet. 2025 Jul 9;16:1611138. doi: 10.3389/fgene.2025.1611138 (PMC12283286; doi:10.3389/fgene.2025.1611138)
Supplement: Supplementary file 1 [file Table1.docx]

**Table S1**

| Primer names | Sequences (5’-3’) | Fragment size |
| --- | --- | --- |
| PATL2-c223-F | CCAGGAGAGGGAGAGGGAT | 305bp |
| PATL2-c223-R | GGGTAGACTGAACATAATAGATGGAA |  |
| PATL2-c877-F | GACATTTTTCTCTCCCCTTTGG | 405bp |
| PATL2-c877-R | CTGGTTGCTTTGCTGCTCA |  |
| PATL2-E10-F | AGAAGCAGGCAGACGAAGAG | 430bp |
| PATL2-E14-R | CCCTTCCTCACAGAGAGCAC |  |
| PATL2-cDNA-RT-E9E12-F | CGCCTGGATGACTACTATTACC | 280bp |
| PATL2-cDNA-RT-E9E12-R | ATACCCGAAGCCTCTGACTG |  |
| PATL2-cDNA-RT-E7E9-F | CACTTTGGACCTCGGCTG | 227bp |
| PATL2-cDNA-RT-E7E9-R | ATCACCCAGTCCTTCTCTTTTC |  |
| GAPDH-cDNA-RT-F | GAGTCAACGGATTTGGTCGT | 225bp |
| GAPDH-cDNA-RT-R | GATCTCGCTCCTGGAAGATG |  |

**Table S1:** Primers were designed for genomic DNA variant validation, cDNA sequencing and qRT-PCR analysis of mRNA.
